# Metabolomic profiling of skeletal muscle in GSDMD-knockout mice reveals distinct metabolic alterations in sepsis-induced myopathy

**DOI:** 10.1515/med-2025-1311

**Published:** 2026-02-24

**Authors:** Yongsheng Zhang, Qinxin Liu, Ligang Xu, Dongfang Wang, Xiangjun Bai, Zhanfei Li, Yukun Liu, Yuchang Wang

**Affiliations:** Division of Trauma Surgery, Emergency Surgery & Surgical Critical, Tongji Trauma Center, Wuhan, P.R. China; Department of Emergency and Critical Care Medicine, Tongji Hospital, Tongji Medical College, Huazhong University of Science and Technology, Wuhan, P.R. China; Department of Plastic and Cosmetic Surgery, Tongji Hospital, Tongji Medical College, Huazhong University of Science and Technology, Wuhan, P.R. China

**Keywords:** GSDMD, sepsis, sepsis-induced muscle myopathy, metabolomic profiling, pyroptosis

## Abstract

**Objective:**

Gasdermin D (GSDMD), a key pyroptosis effector, is implicated in systemic inflammation during sepsis. However, its role in skeletal muscle metabolism remains largely unexplored.

**Methods:**

GSDMD-knockout (GSDMD-KO) and wild-type (WT) mice were used to establish a septic model. Skeletal muscle samples were collected and subjected to non-targeted metabolomic analysis via UHPLC-QE-MS. Multivariate statistical analysis and KEGG pathway enrichment were performed to identify differential metabolites and explore the underlying metabolic alterations.

**Results:**

GSDMD knockout resulted in significant changes in skeletal muscle metabolism, notably in pathways related to taurine and hypotaurine metabolism, amino acid biosynthesis, bile acid biosynthesis, oxidative stress response, and nucleotide metabolism. These alterations suggest that GSDMD regulates energy, amino acid, lipid, and redox metabolism during sepsis. A panel of potential biomarkers was identified, which may contribute to muscle injury and repair.

**Conclusions:**

GSDMD deficiency profoundly alters skeletal muscle metabolic profiles in sepsis. Identified metabolites may serve as diagnostic markers and therapeutic targets for sepsis-associated myopathy, offering insights into GSDMD’s role in muscle metabolism and potential intervention strategies.

## Introduction

Skeletal muscle is one of the largest tissues in the body, playing an indispensable role in maintaining vital functions and overall physiological homeostasis [1]. In addition to participating in crucial biochemical processes such as glucose metabolism, lipid metabolism, and amino acid metabolism, skeletal muscle is essential for maintaining systemic metabolic balance [2]. Skeletal muscle atrophy is a common complication in sepsis survivors, affecting respiratory and motor functions and significantly impairing quality of life and long-term survival [3]. Under septic conditions, in addition to changes in inflammatory mediators [2], 4], skeletal muscle undergoes alterations in a wide range of metabolic products [5], 6]. Therefore, understanding and modulating the regulatory mechanisms of skeletal muscle metabolism is crucial for elucidating the pathophysiological processes of sepsis-associated myopathy.

Gasdermin D (GSDMD) is a key regulator of cell pyroptosis and is often referred to as the “executioner” of pyroptosis [7], 8]. Excessive pyroptosis is considered a critical mechanism in the pathogenesis of inflammatory diseases, including sepsis [9], 10]. During microbial infections, the classical pyroptotic pathway responds to pathogen-associated molecular patterns (PAMPs) and damage-associated molecular patterns (DAMPs), whereas the non-classical pathway responds to cytoplasmic lipopolysaccharides (LPS) from Gram-negative bacteria. In the classical pathway, caspase-1, and in the non-classical pathway, caspase-4/5/11 (caspase-4/5 in humans, caspase-11 in mice), cleave Gasdermin D (GSDMD) into its N- and C-terminal fragments [11]. The N-terminal fragment of GSDMD (GSDMD-N) forms pores on the cell membrane, leading to cell rupture and the release of cytoplasmic contents and inflammatory mediators such as IL-1β and IL-18, triggering an inflammatory response. The N-terminal fragment of GSDMD (GSDMD-N) forms pores on the cell membrane, leading to cell rupture and the release of cytoplasmic contents and inflammatory mediators such as IL-1β and IL-18, triggering an inflammatory response [11]. It has been well established that excessive activation of Gasdermin D contributes to organ dysfunction in sepsis, including lung injury, liver damage, and coagulation disorders, whereas GSDMD knockout exerts a protective effect against multi-organ failure in sepsis [7], [11], [12], [13], [14]. Recent studies suggest that pyroptosis plays a crucial role in the development of skeletal muscle atrophy [15], [16], [17]. Our recent findings also indicate that GSDMD knockout significantly alleviates sepsis-induced skeletal muscle atrophy in mice [18]. However, the specific role of the pyroptosis execution protein GSDMD in the metabolic regulation of skeletal muscle under septic conditions remains unclear.

Over the past few decades, metabolomics has emerged as a powerful tool for elucidating metabolic pathways and identifying disease-related biomarkers. Metabolomics systematically investigates the quantitative and qualitative changes of all metabolites within a biological system, providing a comprehensive understanding of metabolic regulation [19]. This approach holds immense potential in early disease diagnosis and therapeutic development, particularly in identifying metabolic disturbances and biomarkers [20]. This study aims to explore the potential impact of GSDMD knockout on skeletal muscle metabolism and identify potential biomarkers through metabolomic analysis. By characterizing the metabolomic profiles of skeletal muscle in GSDMD-knockout mice, we seek to gain deeper mechanistic insights into the protective role of GSDMD knockout in sepsis-associated muscle dysfunction. Additionally, by identifying potential metabolic biomarkers.

## Materials and methods

### Animals

All animal experiments in this study were approved by the Animal Welfare and Ethics Committee of Tongji Hospital (Ethics Approval No.: TJH-201809002). Male wild-type C57BL-6 and GSDMD−/− mice (8–12 weeks old) were purchased from Beijing Vital River Laboratory Animal Technology Co., Ltd. Genotyping was performed using polymerase chain reaction (PCR) amplification of tail-tip DNA samples. Mice were housed in a specific pathogen-free (SPF) facility under a 12-h light/dark cycle. Standard rodent chow (Jiangsu Synergy Pharmaceutical Bioengineering Co., Ltd., China) and water were provided *ad libitum*. The cecal ligation and puncture (CLP) surgery was performed by experienced surgeons in male mice (22–26 g body weight) according to established guidelines. Mice were euthanized at designated time points post-surgery using a randomized and blinded approach, and tissue samples were collected for analysis. The methodology is consistent with our previous studies [18].

### Establishment of the CLP mouse model

CLP surgery was performed in male mice (22–26 g body weight) following standardized procedures [21]. Mice were euthanized at different postoperative time points using a randomized and blinded approach, and tissue samples were collected accordingly. The biological assays performed on the collected mouse samples have been reported in our previous study [18].

### Tissue sample collection

On the fourth day after CLP surgery, skeletal muscle tissue samples were collected from both the control and GSDMD-knockout groups. Following euthanasia, skeletal muscle tissues were rapidly excised and rinsed with physiological saline to remove residual blood and contaminants. The collected tissue samples were divided into two parts: one for molecular biology experiments, as previously published [18], and the other for metabolomic analysis to investigate the impact of GSDMD knockout on skeletal muscle metabolism.

### Untargeted metabolomic analysis

Gastrocnemius muscle samples (25 mg) were obtained and mixed with 500 μL extraction solution (methanol: acetonitrile: water=2:2:1) containing an isotope-labeled internal standard mixture. The samples were vortexed for 30 s, sonicated in an ice bath for 10 min, and incubated at −40 °C for 1 h to precipitate proteins. After incubation, the samples were centrifuged at 12,000 rpm for 15 min at 4 °C, and the supernatant was transferred to 2 mL glass vials for analysis using ultra-high-performance liquid chromatography coupled with high-resolution mass spectrometry (UHPLC-QE-MS).

Quality control (QC) samples were prepared by pooling equal volumes of supernatants from all experimental samples. The final dataset, containing peak numbers, sample names, and normalized peak areas, was imported into the SIMCA 16.0.2 multivariate analysis software (Sartorius Stedim Data Analytics AB, Umea, Sweden) for statistical analysis. Variable importance in projection (VIP) values for the first principal component was obtained using orthogonal partial least squares-discriminant analysis (OPLS-DA), summarizing each variable’s contribution to the model. Metabolites with VIP>1 and p<0.05 (Student’s *t*-test) were considered significantly altered. Furthermore, pathway enrichment analysis was performed using commercial databases, including the Kyoto Encyclopedia of Genes and Genomes (KEGG, http://www.genome.jp/kegg/) and MetaboAnalyst (http://www.metaboanalyst.ca/).

### Data processing and statistical analysis

After metabolomic profiling, a comprehensive data processing and analysis workflow was implemented to ensure high data quality and robust statistical inference. Preprocessing steps included noise filtering, data alignment, and imputation of missing values to address technical variability and maintain consistency across samples. Multivariate statistical analyses, including principal component analysis (PCA) and partial least squares-discriminant analysis (PLS-DA), were then performed to assess metabolic differences between experimental groups and identify pathways associated with GSDMD knockout.

For univariate statistical testing, appropriate hypothesis tests were applied, and all resulting p-values were adjusted using the Benjamini–Hochberg false discovery rate (FDR) correction to control for multiple comparisons. Metabolites with adjusted q-values<0.05 were considered statistically significant.

## Results

### Sample preparation and principal component analysis

**Figure 1:**
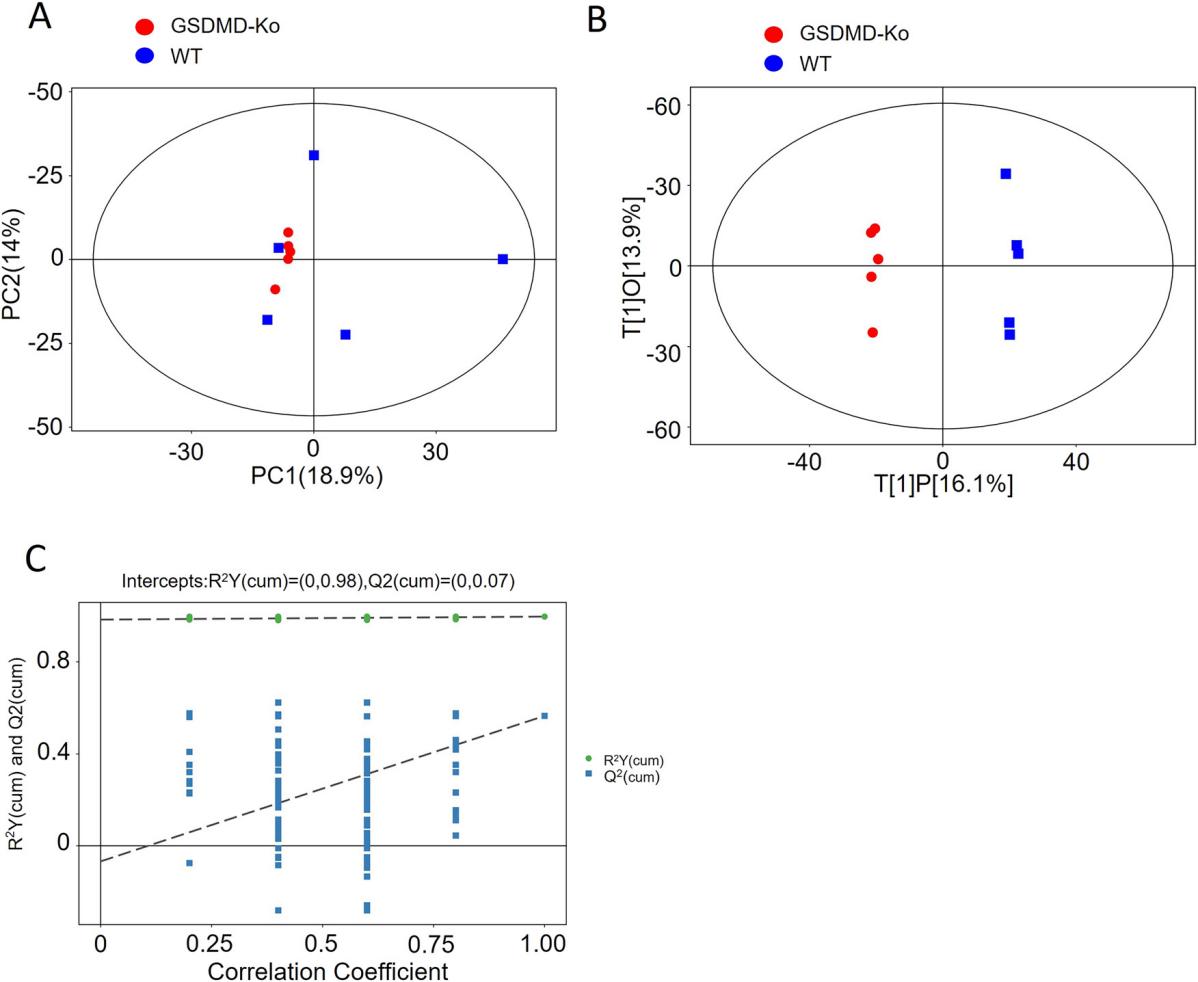
Multivariate statistical analysis of metabolomic profiles. (A) PCA score scatter plot for the total dataset. The horizontal axis represents PC1 (principal component 1) scores, and the vertical axis represents PC2 (principal component 2) scores. Each data point represents an individual sample, with different colors and shapes indicating different experimental groups. Samples closer together indicate higher similarity in metabolite composition, while greater distance reflects larger differences in overall metabolic profiles. (B) OPLS-DA score scatter plot for group GSDMD KO vs. WT. The horizontal axis represents t[1]P, the predictive component score of the first principal component, showing inter-group differences. The vertical axis represents t[1]O, the orthogonal component score, indicating intra-group variation. Each data point represents a sample, with different shapes and colors representing distinct experimental groups. The greater horizontal separation between samples indicates larger inter-group differences, while closer vertical alignment signifies better intra-group reproducibility. (C) Permutation test of OPLS-DA model for group GSDMD-KO vs. WT. The horizontal axis represents the permutation retention rate of the permutation test, reflecting the proportion consistent with the original model’s Y-variable order (the point at which the permutation retention rate equals 1 corresponds to the R Y and Q values of the original model). The vertical axis represents the values of R Y or Q. Green dots represent R Y values obtained from the permutation test, while blue squares represent Q values from the same test. The two dashed lines represent regression lines fitted to all R Y and Q values in the models.

A total of 10 skeletal muscle samples were analyzed, comprising two groups: the GSDMD-KO group and the WT group, each with five biological replicates. Metabolomic analysis based on Q Exactive Orbitrap (QE) was performed. The data were log-transformed (LOG) and centered (CTR) using SIMCA software (V16.0.2, Sartorius Stedim Data Analytics AB, Umea, Sweden), followed by automatic modeling analysis. The PCA score plot demonstrated that all samples fell within the 95 % confidence interval (Hotelling’s T-squared ellipse) (Figure 1A). The OPLS-DA score plot indicated a highly significant differentiation between the two groups, with all samples falling within the 95 % confidence interval (Hotelling’s T-squared ellipse) (Figure 1B). The permutation test results for the OPLS-DA model demonstrated its robustness (Figure 1C).

### Extraction and analysis of differential metabolites

**Figure 2:**
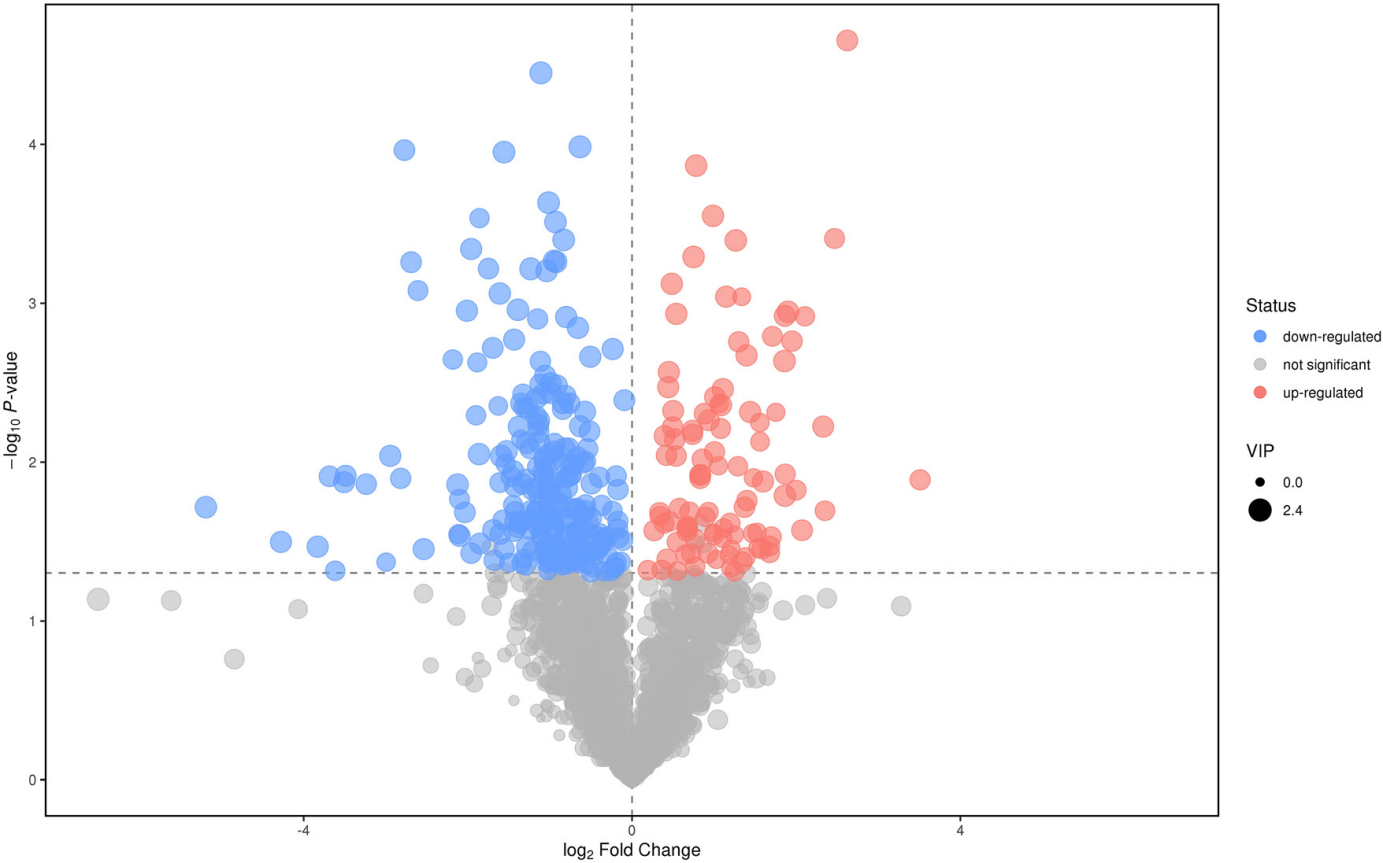
The volcano plot displays all metabolites detected in the experiment. Each point represents a metabolite. The horizontal axis represents the fold change in the comparison of the two groups (in logarithm base 2), and the vertical axis represents the negative logarithm (base 10) of the p-value from Student’s *t*-test. The size of each point corresponds to the VIP value from the OPLS-DA model. Significantly upregulated metabolites are shown in red, significantly downregulated metabolites in blue, and non-significantly different metabolites in gray.

After FDR correction, the PCA, OPLS-DA, and PLS-DA models identified 100 upregulated and 233 downregulated features in the GSDMD-KO-sepsis group compared with the WT-sepsis group under negative ion mode (q<0.05, VIP>1) (Figure 2). Detailed information is available in Supplementary Table 1.

### Hierarchical clustering analysis of differential metabolites

**Figure 3:**
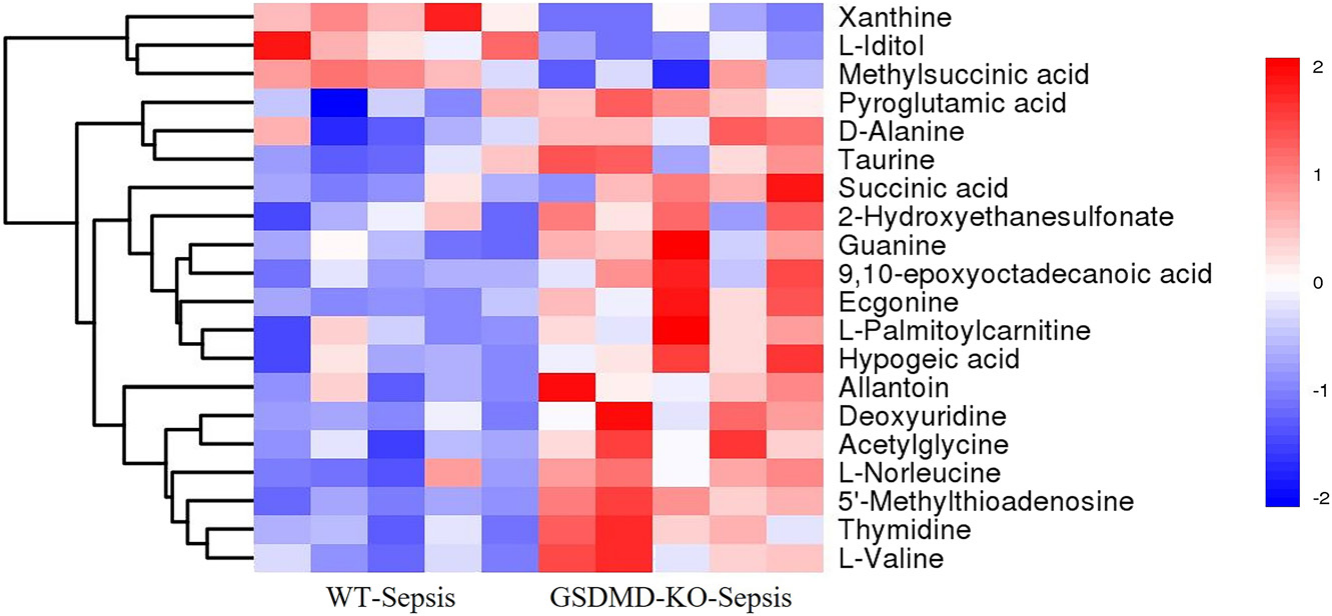
Heatmap of hierarchical clustering analysis for group GSDMD-KO vs. WT. The horizontal axis represents different experimental groups, and the vertical axis represents differentially expressed metabolites in the comparison. The color blocks at different positions represent the relative expression levels of corresponding metabolites. Red indicates high expression in the respective group, while blue indicates low expression.

The quantified values of differential metabolites were analyzed using Euclidean distance matrices and hierarchical clustering with complete linkage, visualized through heatmaps (Figure 3). This analysis revealed that GSDMD knockout markedly altered metabolite profiles in septic mice. Specifically, metabolites such as Xanthine, L-Iditol, and Methylsuccinic acid were significantly upregulated in the GSDMD-KO group compared to the WT group. Conversely, a range of metabolites including Pyroglutamic acid, d-Alanine, Taurine, Succinic acid, and l-Valine were downregulated (Supplementary Table 2). These findings suggest that GSDMD deficiency modulates key metabolic pathways involved in purine metabolism, amino acid metabolism, and energy production.

### Correlation analysis

**Figure 4:**
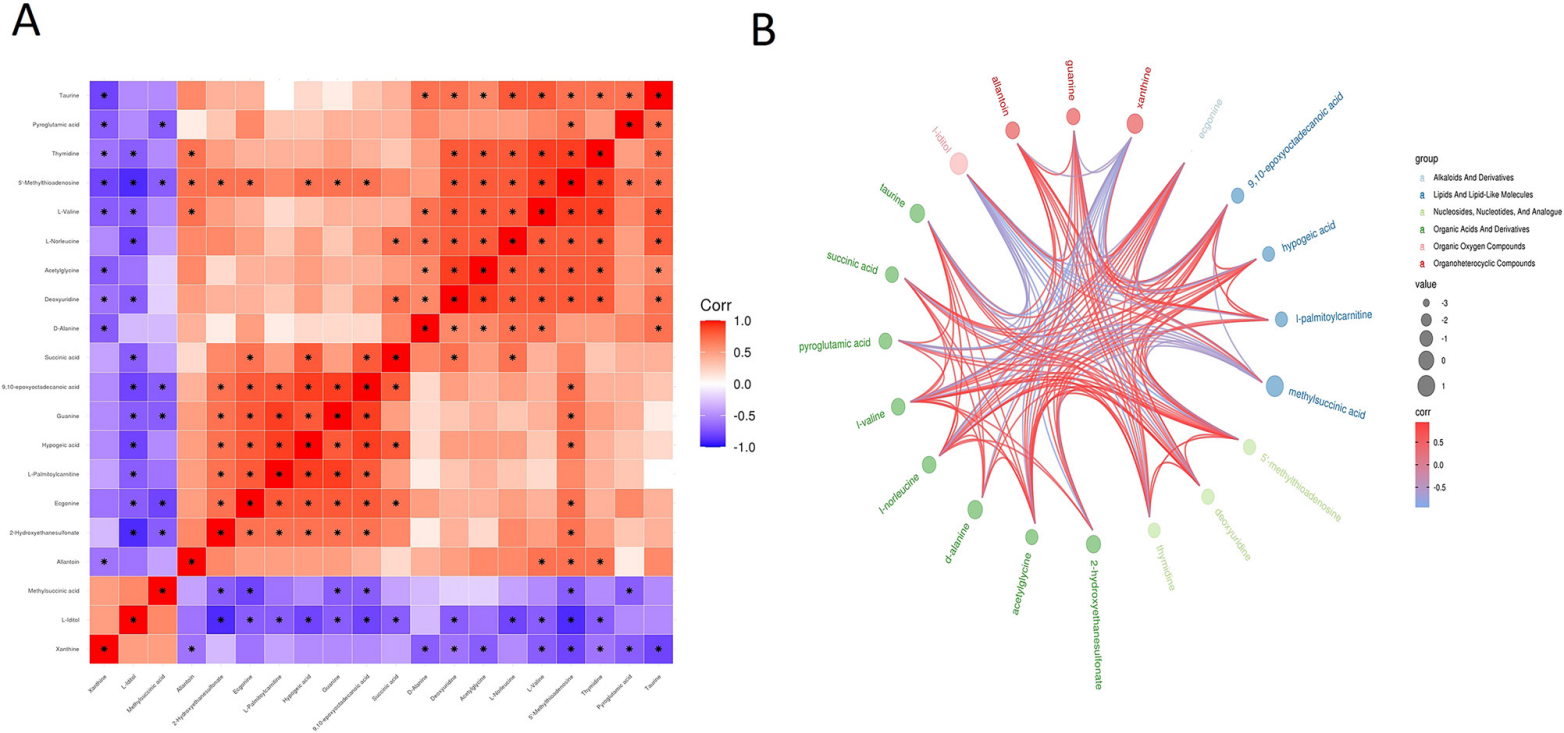
Correlation analysis of differentially expressed metabolites between GSDMD-KO and WT groups. (A) Heatmap of correlation analysis for group GSDMD-KO vs. WT. The horizontal and vertical axes represent differentially expressed metabolites in the comparison. The color blocks at different positions represent the magnitude of the correlation coefficients between corresponding metabolites. Red indicates a positive correlation, blue indicates a negative correlation and darker colors signify stronger correlations. Significantly correlated pairs are marked with asterisks (✳). (B) Chord plot analysis for group GSDMD-KO vs. WT. The size of each point represents the LOG_FOLDCHANGE value, with larger points corresponding to larger LOG_FOLDCHANGE values. Point colors represent the classification of differentially expressed metabolites. The lines between points represent the magnitude of the correlation coefficient values for corresponding metabolites.

Metabolites often exhibit significant intercorrelations, and divergent changes among structurally or functionally related metabolites frequently reflect underlying biological processes. In our analysis, several metabolites involved in purine metabolism and energy production (e.g., from xanthine to methylsuccinic acid) showed positive correlations, suggesting potential coordinated regulation of nucleotide turnover and mitochondrial activity. Conversely, multiple amino acids and related intermediates (e.g., from pyroglutamic acid to l-valine) displayed negative correlations, which may be associated with altered amino acid utilization and protein turnover following GSDMD deletion. Another group of metabolites related to antioxidant defense pathways also exhibited positive correlations, implying that GSDMD-KO mice may have enhanced antioxidant capacity (Figure 4A).

Furthermore, the chord diagram provided an intuitive visualization of the relationships between metabolite classes and their directional changes. By categorizing the differential metabolites and calculating their Spearman correlations, we uncovered intricate interactions among purine metabolism, amino acid metabolism, and antioxidant pathways (Figure 4B). These findings underscore the potential role of GSDMD in orchestrating multiple metabolic networks under septic conditions, particularly those involved in oxidative stress regulation and muscle homeostasis.

### Pathway clustering analysis

**Figure 5:**
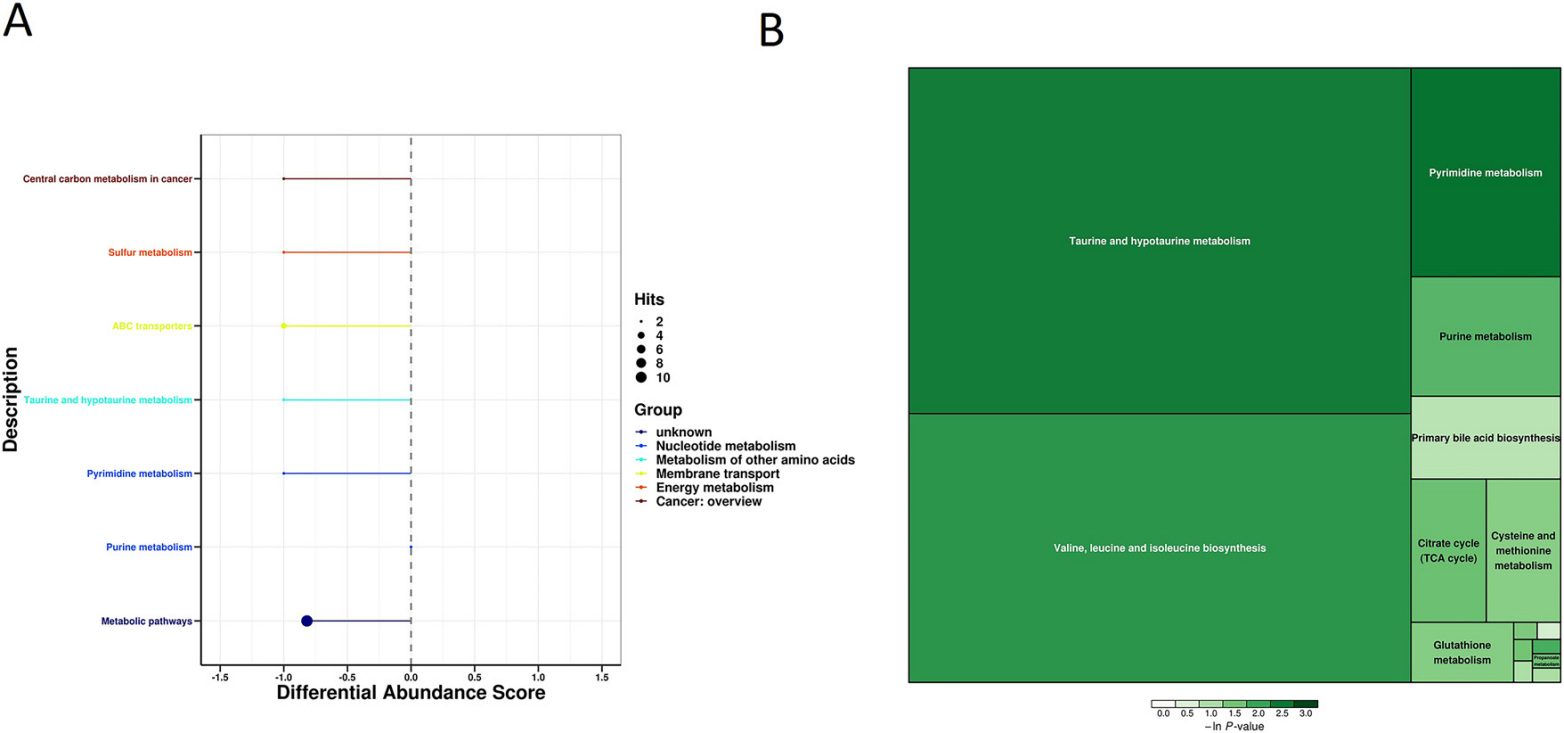
KEGG pathway differential abundance and enrichment analysis. (A) Differential abundance analysis of KEGG pathways. Different colors of the pathways represent different metabolic categories. Line segments indicate whether the pathway is upregulated or downregulated, with positive values indicating an overall upregulation and negative values indicating an overall downregulation. The size of the endpoints of the line segments represents the number of annotated substances in that pathway. (B) Metabolic pathway enrichment analysis of differential metabolites. The results of metabolic pathway analysis are displayed as a rectangular treemap. Each square represents a metabolic pathway, with square size indicating the impact factor in topological analysis (a larger size corresponds to a larger impact factor). The color of the squares represents the p-value from the enrichment analysis (natural logarithm, –ln(p)); darker colors indicate smaller p-values, signifying greater enrichment significance.

After analyzing the changes in skeletal muscle metabolites in GSDMD-KO mice, we studied enriched pathways to construct enrichment diagrams. KEGG pathway differential abundance analysis indicated that GSDMD knockout primarily affected pathways such as Lysine degradation, metabolic pathways, choline metabolism in cancer, Necroptosis, Glycerophospholipid metabolism, Sphingolipid metabolism, ABC transporters, Retrograde endocannabinoid signaling, Sphingolipid signaling pathway, and Lysine degradation (Figure 5A). Enrichment analysis of differential metabolites’ metabolic pathways suggested that GSDMD knockout mainly influenced Taurine and hypotaurine metabolism, Valine, leucine, and isoleucine biosynthesis, Pyrimidine metabolism, purine metabolism, primary bile acid biosynthesis, Citrate cycle (TCA cycle), and Cysteine and methionine metabolism. The emphasized metabolic pathways in the enrichment analysis revealed significant alterations in energy, amino acid metabolism, as well as transfer-degradation-cycling processes (Figure 5B).

## Discussion

In this study, we investigated the skeletal muscle responses of WT and GSDMD−/− mice to sepsis induction using metabolomics techniques. Our results revealed that GSDMD−/− promotes taurine and hypotaurine metabolism, amino acid biosynthesis, primary bile acid biosynthesis, and antioxidant stress capacity in skeletal muscles. Moreover, it regulates nucleotide metabolism. Based on our findings, GSDMD knockout significantly impacts the metabolism of mouse skeletal muscles, involving multiple pathways such as energy metabolism, amino acid metabolism, oxidative stress, and lipid metabolism. This suggests a potentially significant role of GSDMD in skeletal muscle metabolism regulation.

Enhanced oxidative stress is a critical mechanism underlying skeletal muscle wastage and atrophy during sepsis [22]. Increased reactive oxygen species and oxidative stress molecules in sepsis-induced myopathy can lead to oxidative damage in cells, including lipid peroxidation, protein oxidation, and DNA oxidation, resulting in muscle dysfunction, structural impairment, and cell death [23]. Furthermore, oxidative stress can activate inflammatory responses and inflammatory cell activation, forming a vicious cycle that exacerbates muscle damage [23], 24]. Taurine, a sulfur-containing non-protein amino acid, exists in the body in a free state. Although it is not directly involved in protein synthesis, it exhibits anti-inflammatory and antioxidant properties [25]. Numerous studies have reported decreased taurine levels in sepsis and its depletion in skeletal muscles has been shown to accelerate muscle aging, alter muscle metabolism, increase muscle atrophy, disrupt myofiber integrity, cause calcium homeostasis imbalance, and shorten lifespan. Taurine supplementation has been shown to improve muscle regeneration in malnourished muscles, enhance glucose homeostasis, prevent muscle damage due to overuse, and alleviate age-related muscle loss [26], [27], [28]. As reflected in our results, Taurine and hypotaurine levels were elevated in the GSDMD-KO group, along with Uracil and Glutathione. Uracil and Glutathione, as antioxidants, are indicators of the intensity of oxygen radicals [29], 30]. The increase in their levels suggests that GSDMD-KO enhances antioxidant stress levels in muscles, providing a mechanistic explanation for its role in improving sepsis-induced muscle wastage and atrophy.

Amino acids, serving as building blocks for proteins, play pivotal roles in skeletal muscle cells. Sepsis not only reduces amino acid uptake by skeletal muscles [31] but also induces changes in amino acid transporters and leucine signaling pathways [32]. Gln and/or leucine can reduce sepsis-induced muscle degradation and promote muscle-specific gene expression. During sepsis, leucine treatment alone has a more significant effect on maintaining muscle quality [33]. Our study indicated that GSDMD knockout significantly affected the levels of several amino acids, particularly valine, leucine, isoleucine biosynthesis, and methionine metabolism, which are involved in protein synthesis and repair capacity. The involvement of purine and pyrimidine metabolism in sepsis-induced myopathy remains unclear. GSDMD-KO mice showed a significant decrease in inosine and guanosine levels, while xanthine levels were significantly increased. This change might be related to the influence on xanthine oxidase activity. Inhibition of xanthine oxidase has been shown to improve muscle contractility [34], 35], providing an additional interpretation for enhanced muscle strength in GSDMD-KO mice.

Succinic acid, a key intermediate in the citric acid cycle, has been considered an inflammatory mediator in metabolism [36]. However, studies have also shown that succinic acid induces skeletal muscle fiber transformation through the SUNCR1 signaling pathway, enhancing mouse skeletal muscle endurance, myosin heavy chain I expression, aerobic enzyme activity, oxygen consumption, and mitochondrial biogenesis [37]. In our study, GSDMD-KO mice exhibited significantly elevated levels of succinic acid, and further exploration is needed to decipher the underlying regulatory mechanisms. Additionally, GSDMD might play a role in regulating skeletal muscle lipid metabolism. Lipids not only serve as an energy source in muscle cells but also participate in regulating cell membrane structure and signal transduction. Our experimental results demonstrated that GSDMD knockout significantly influenced the levels of several lipid metabolites. By affecting cell membrane integrity and inflammation response, GSDMD might regulate lipid synthesis and degradation, thereby influencing skeletal muscle lipid metabolism.

In summary, we uncovered that GSDMD potentially functions in skeletal muscle metabolism regulation by modulating oxidative stress, subsequently impacting critical metabolic pathways such as energy metabolism, amino acid metabolism, and lipid metabolism. These potential biomarkers may play vital regulatory roles in skeletal muscle metabolism and possess significant physiological and clinical application potential. These markers could provide new insights into the pathological processes of sepsis-induced myopathy. Quantitative and qualitative analysis of these markers can deepen our understanding of the pathogenesis and pathological processes of metabolic diseases, offering new leads for early diagnosis and treatment of related diseases. Furthermore, these potential biomarkers could serve as novel indicators for the early diagnosis and prevention of sepsis-related diseases. This would contribute to reducing the incidence of metabolic diseases and alleviating the severity of these conditions. Finally, these potential biomarkers could offer new targets and strategies for the treatment of sepsis-induced myopathy. Our findings indicate that GSDMD plays a pivotal role in skeletal muscle metabolism regulation, and these GSDMD-related biomarkers might be closely related to its function. Therefore, a therapeutic intervention targeting these markers could potentially regulate skeletal muscle metabolism, paving the way for personalized treatment and precision medicine in metabolic diseases.

## Limitations and future perspectives

In this study, we have preliminarily elucidated the role of GSDMD in regulating skeletal muscle metabolism and identified a series of potential biomarkers. However, several limitations remain. First, although we employed a specific GSDMD knockout mouse model to investigate its function, gene knockout may also affect other related signaling pathways, making it difficult to completely rule out confounding factors. Second, while metabolomics analysis enables comprehensive profiling of numerous metabolites, the limited sample size may affect the overall coverage and reliability of the results.

To further explore the role of GSDMD in skeletal muscle metabolism, future studies should incorporate more precise genetic and molecular biology approaches, such as gene knockout/knock-in techniques and specific activation or inhibition of relevant signaling pathways, to validate the current findings. Additionally, increasing the sample size and enhancing the statistical rigor of metabolomics data analysis will improve the robustness and reproducibility of the results.

The relatively small sample size in this study (n=5 per group) may restrict the detection of metabolites with subtle effects. Nevertheless, key differential metabolites, including taurine, xanthine, and succinate, exhibited fold changes greater than 1.7 and Cohen’s d effect sizes ranging from 1.2 to 1.5, indicating substantial effect sizes. Post-hoc power analysis demonstrated that, at the current sample size, the statistical power for detecting these metabolites reached ≥80 %. Still, future investigations with larger cohorts are warranted to further validate the reliability and reproducibility of these findings. Moreover, targeted metabolomics validation (e.g., targeted LC–MS/MS) of these key metabolites in larger clinical sepsis cohorts will be instrumental in assessing their consistency and potential utility as biomarkers for metabolic disease diagnosis, prevention, and treatment.

Furthermore, validation of the identified biomarkers in clinical samples, along with clinical trials and case-control studies, will be critical to evaluate their translational potential in diagnosing, preventing, and treating metabolic diseases. By implementing these improvements, we are confident that future research will deepen the understanding of GSDMD’s role in skeletal muscle metabolic regulation and provide novel insights for precision medicine and personalized therapies in related metabolic disorders.

## Conclusions

This study reveals the critical role of GSDMD in regulating skeletal muscle metabolism and identifies a series of potential biomarker candidates associated with GSDMD deficiency through metabolomics analysis. These findings underscore the importance of GSDMD in skeletal muscle metabolic reprogramming and offer new insights into the pathological mechanisms of septic myopathy and related metabolic disorders. The identified metabolites represent promising biomarker candidates for future research; however, their diagnostic and therapeutic value requires further clinical validation and functional studies before they can be applied to the prevention, diagnosis, and management of metabolic diseases.

## Supplementary Material

Supplementary Material

Supplementary Material
